# The original anticonvulsant meta-Cl-benzhydrylurea (m-CL-BHM) induces of the liver monooxygenase system and stimulates the neuroimmune response in behavioral disorders of alcoholic origin

**DOI:** 10.1192/j.eurpsy.2022.2120

**Published:** 2022-09-01

**Authors:** Т. Shushpanova, T. Novozheeva, E. Gutkevich, A. Pustovaya, N. Garganeeva, M. Belousov, A. Podyablonsky, E. Markova, E. Knyazeva, O. Shushpanova, N. Kolomiets, S. Safronov, R. Boev

**Affiliations:** 1 Mental Health Research Institute of Tomsk National Investigation MedicalCenter of Russian Academy of Sciences, Clinical Psychoneuroimmunology And Neurobiology, Tomsk, Russian Federation; 2 Mental Health Research Institute Tomsk National Research Medical Center Russian Academy of Sciences, Department Of Endogenous Disorders, Tomsk, Russian Federation; 3 Tomsk state University, Faculty Of Psychology, Tomsk, Russian Federation; 4 Siberian State Medical University, Department Of General Medical Practice And Outpatient Therapy, Lenina Avenu, Russian Federation; 5 Siberian State Medical University, Department Of Pharmaceutical Analysis, Tomsk, Russian Federation; 6 National Research Tomsk Polytechnic University, Research School Of The Chemical And Biomedical Technologies, Tomsk, Russian Federation; 7 State Research Institute of Fundamental and Clinical Immunology, Neuroimmunology, Novosibirsk, Russian Federation; 8 National Research Tomsk Polytechnic University, Institute Of Physics Of High Technologies, Tomsk, Russian Federation; 9 Mental Health Scientific Center, Child Psychiatry, Moscow, Russian Federation; 10 Scientific Research Institute of Pharmacology and Regenerative Medicine named after E.D. Goldberg, Physiology And Pharmacology, Tomsk, Russian Federation

**Keywords:** anticonvulsant, cytochrome, receptor, homeostasis, immune function

## Abstract

**Introduction:**

The anticonvulsant m-Cl-BHM is promising for the pathogenetically directed thrapy does not cause negative effects.

**Objectives:**

Investigation of the effect of m-Сl-BHM on “immunochemical homeostasis” in rats with experimental alcoholism.

**Methods:**

m-Cl-BHM was injected at a dose of 100 mg/kg (1/20 LD50) for 5 and 30 days into the stomach of male Wistar rats who preferred alcohol according to the screening conditions and kept for 10 months. in free access to a 15% ethanol solution, which made up the group of “heavy drinkers” (HD). Phenobarbital was administered at a dose of 25 mg/kg (1/20 LD50).

**Results:**

The features of the monooxygenase system of cytochrome P450 of the liver and ECT in the lymphoid organs of rats were studied at different periods of administration of m-CL-BHM -5 and 30 days. to HD rats. m-CL-BHM has an inducing effect on the monooxygenase system of the liver, causes phase changes in the lymphoid organs and ECT. Long-term administration of m-CL-BHM caused a depletion of the cellular composition of lymphoid organs, a decrease in ECT of spleen cells and peritoneal exudate, these changes were less pronounced compared with phenobarbital. The activation of the immune system inversely regulates the production of enzymes of the cytochrome system, since the concentration of low molecular weight targets is sharply reduced with the help of antibodies. m-Cl-BHM metabolites conjugated to endogenous macromolecules form a full-fledged stimulus for the immune system.

**Conclusions:**

Neuroimmune response to the introduction of m-CL-BHM is significant in behavioral disorders associated with alcoholism and the correction of this condition.

**Disclosure:**

No significant relationships.

